# iTRAQ-Based Proteomics Reveals Gu-Ben-Fang-Xiao Decoction Alleviates Airway Remodeling via Reducing Extracellular Matrix Deposition in a Murine Model of Chronic Remission Asthma

**DOI:** 10.3389/fphar.2021.588588

**Published:** 2021-06-14

**Authors:** Qiongqiong Xing, Yannan You, Xia Zhao, Jianjian Ji, Hua Yan, Yingmei Dong, Lishun Ren, Yuanyuan Ding, Shuting Hou

**Affiliations:** ^1^ Affiliated Hospital of Nanjing University of Chinese Medicine, Nanjing, China; ^2^ Pediatric Institution of Nanjing University of Chinese Medicine, Nanjing, China; ^3^ Jiangsu Key Laboratory of Pediatric Respiratory Disease, Nanjing, China

**Keywords:** GU-BEN-FANG-XIAO decoction, airway remodeling, extracellular matrix deposition, chronic remission, asthma

## Abstract

Airway remodeling is a primary pathological feature of asthma. The current therapy for asthma mainly targets reducing inflammation but not particularly airway remodeling. Therefore, it is worthwhile to develop alternative and more effective therapies to attenuate remodeling. Gu-Ben-Fang-Xiao Decoction (GBFXD) has been used to effectively and safely treat asthma for decades. In this study, GBFXD regulated airway inflammation, collagen deposition, and the molecules relevant to airway remodeling such as Vimentin, α-SMA, hydroxyproline, and E-cadherin in chronic remission asthma (CRA) murine model. Proteomic analysis indicated that the overlapping differentially expressed proteins (DEPs) (Model/Control and GBFXD/Model) were mainly collagens and laminins, which were extracellular matrix (ECM) proteins. In addition, the KEGG analysis showed that GBFXD could regulate pathways related to airway remodeling including ECM-receptor interactions, focal adhesion, and the PI3K/AKT signaling pathway, which were the top three significantly enriched pathways containing the most DEPs for both Model/Control and GBFXD/Model. Further validation research showed that GBFXD regulated reticulon-4 (RTN4) and suppressed the activation of the PI3K/AKT pathway to alleviate ECM proteins deposition. In conclusion, our findings indicate that GBFXD possibly regulate the PI3K/AKT pathway via RTN4 to improve airway remodeling, which provides a new insight into the molecular mechanism of GBFXD for the treatment of CRA.

## Introduction

Asthma is a heterogeneous disease of high prevalence worldwide, characterized by respiratory symptoms such as recurrent wheeze, coughing, and shortness of breath caused by lumen narrowing and airflow obstruction (Camoretti-Mercado and Lockey, 2020). Epidemiological studies predict that the number of patients with asthma will reach 400 million by 2025 (Voskamp et al., 2020). Asthma is the most prevalent chronic respiratory disease in children, affecting approximately 10% of the general population and up to 20% of children, with approximately a 50% increase in prevalence each decade (Croisant, 2014). Inhaled corticosteroids (ICS) has been the most effective anti-inflammatory medication in controlling symptoms and improving the quality of life for patients with asthma (Costello and Cushen, 2020). However, the effect of ICS on attenuating airway remodeling remains controversial (Halwani et al., 2016; Yap et al., 2019) and the current therapies for asthma do not particularly target airway remodeling (Prakash et al., 2017). The underlying mechanism of airway remodeling in asthma remains unclear, but chronic inflammation and/or mechanical stresses from repeated bronchoconstriction independent of inflammation play vital roles (Grainge et al., 2011; Prakash et al., 2017). Importantly, airway remodeling may occur early during asthma and is also related to airway hyperresponsiveness (AHR) (Jendzjowsky and Kelly, 2019). Therefore, developing alternative and more effective therapies to attenuate airway inflammation and remodeling is essential and urgent.

Traditional Chinese medicine (TCM) formulae are usually composed of one or two medicinal herbs as the principal component and the others act as adjuvants to enhance the effects (Tao et al., 2017). The therapeutic efficacy of TCM formulae is widely accepted to treat disease from a holistic point of view. High-throughput proteomic analysis of the functional proteins can help clarify the complex mechanisms of TCM. Gu-Ben-Fang-Xiao Decoction (GBFXD), an empirical formula prescribed by a famous Chinese professor Jiang Yuren, contains 11 herbs (Table 1) and has been used to treat pediatric asthma for decades. Previous clinical studies in which the decoction obtained by the above mentioned herbs were administrated for children orally once a day have confirmed the effectiveness and safety in reducing asthma recurrence (Yuan et al., 2010). Our previous research demonstrated that GBFXD could modulate the microbiota-acetate-regulatory T cell (Treg) axis (Dong et al., 2020), downregulate the asthma susceptibility gene *ORMDL3* (Huang et al., 2016), and suppress endoplasmic reticulum (ER) stress responses (Lu et al., 2016) in a murine model of chronic remission asthma (CRA). In addition, GBFXD has been shown to regulate cholesterol transport, activate complement factors (Xing et al., 2019), and inhibit macrophage polarization (Liu et al., 2019) in a murine model of chronic persistent asthma (CPA); however, little is known regarding the mechanism of GBFXD on airway remodeling in CRA model.

**TABLE 1:** Composition of GBFXD.

Component	Family	Part	Voucher number	Weight (g)
Astragalus mongholicus Bunge (Zhi huang qi)	Leguminosae	Root	NZY-Zhao-2018001	15
Codonopsis pilosula (Franch.) Nannf. (Dang Shen)	Campanulaceae	Root	NZY-Zhao-2018002	10
Poria cocos (Schw.) Wolf. (Fu Ling)	Polygonaceae	Sclerotia	NZY-Zhao-2018003	10
Atractylodes macrocephala Koidz. (Bai Zhu)	Composite	Rhizome	NZY-Zhao-2018004	10
Citrus reticulata Blanco (Chen Pi)	Rutaceae	Peel	NZY-Zhao-2018005	6
Saposhnikovia divaricata (Turcz.) Schischk. (Fang Feng)	Umbelliferae	Root	NZY-Zhao-2018006	3
Cryptotympana pustulata Fabricius (Chan Tui)	Cicadidae	Slough	NZY-Zhao-2018007	6
Magnolia denudata Desr (Xin Yi)	Magnoliaceae	Bud	NZY-Zhao-2018008	6
Schisandra chinensis (Turcz.) Baill. (Wu Wei Zi)	Magnoliaceae	Fruit	NZY-Zhao-2018009	6
Ostrea gigas Thunberg (Duan Mu Li)	Ostreidae	Shell	NZY-Zhao-2018010	15
Glycyrrhiza uralensis Fisch. (Gan Cao)	Leguminosae	Rhizome and root	NZY-Zhao-2018011	3

The structural changes of the airway wall known as airway remodeling, which may explain persistent airflow obstructions and recurrent attacks in some asthmatic patients, can occur early in childhood and increase the risk of advancing into clinical asthma (Bonato et al., 2018). Recent studies reported that airway remodeling in asthmatic children could also arise independently on inflammation (Baraldo et al., 2011), thus much attention should be paid to the prevention and treatment of airway remodeling. Increased deposition of ECM proteins in the reticular basement membrane region, lamina propria, and submucosa is a feature of asthmatic airways and facilitates the airway wall thickening (Hough et al., 2020). Meantime, the molecules such as collagen, fibronectin related to ECM are used to confirm the airway modeling. Collagen is the most abundant component of ECM in the lung and a potential marker of remodeling in asthma (Ojiaku et al., 2017). There is an increase in the deposition of collagen I and collagen III in asthma by bronchial biopsies (Benayoun et al., 2003; Chakir et al., 2003). In addition, fibronectin is the ECM adhesion protein and increased fibronectin level of the airway wall smooth muscle in fatal asthma was reported (Araujo et al., 2008). Emerging studies reported that basement membrane thickening is still present even in clinical and complete remissions (van den Toorn et al., 2001; Broekema et al., 2011). Our previous study reported GBFXD reduced airway remodeling by Masson staining in a murine model of CRA (Huang et al., 2016), but the mechanism remains unknown. Carpaij et al. declared consistent findings that inflammatory markers were elevated in asthma remission compared to those in the healthy controls, and were lower compared to those in persistent asthma cases (Carpaij et al., 2019). Similarly, in previous study we established the murine models of CPA and CRA, airway inflammation, epithelial goblet cells, and AHR were more severe in the CPA model than those in the CRA model (Liu et al., 2019). Considering the differences between CPA and CRA as well as multiple TCM targets, we intend to confirm differentially expressed proteins (DEPs) after GBFXD treatment and further elucidate the possible mechanisms of GBFXD in CRA by iTRAQ-based proteomics.

## Materials and Methods

### GBFXD Preparation

All herbs were purchased from the Affiliated Hospital of Nanjing University of Chinese Medicine, identified by Professor Shengjin Liu from Nanjing University of Chinese Medicine, and deposited into the Herbarium of Traditional Chinese Medicine, Nanjing University of Chinese Medicine (Table 1). The herbs of GBFXD were mixed, decocted as previously described (Dong et al., 2020), and stored with a final concentration of 3 g/ml of crude herbs for further use.

### Components of GBFXD Determined by UPLC-ESI/LTQ-Orbitrap-MS

**FIGURE 1:**
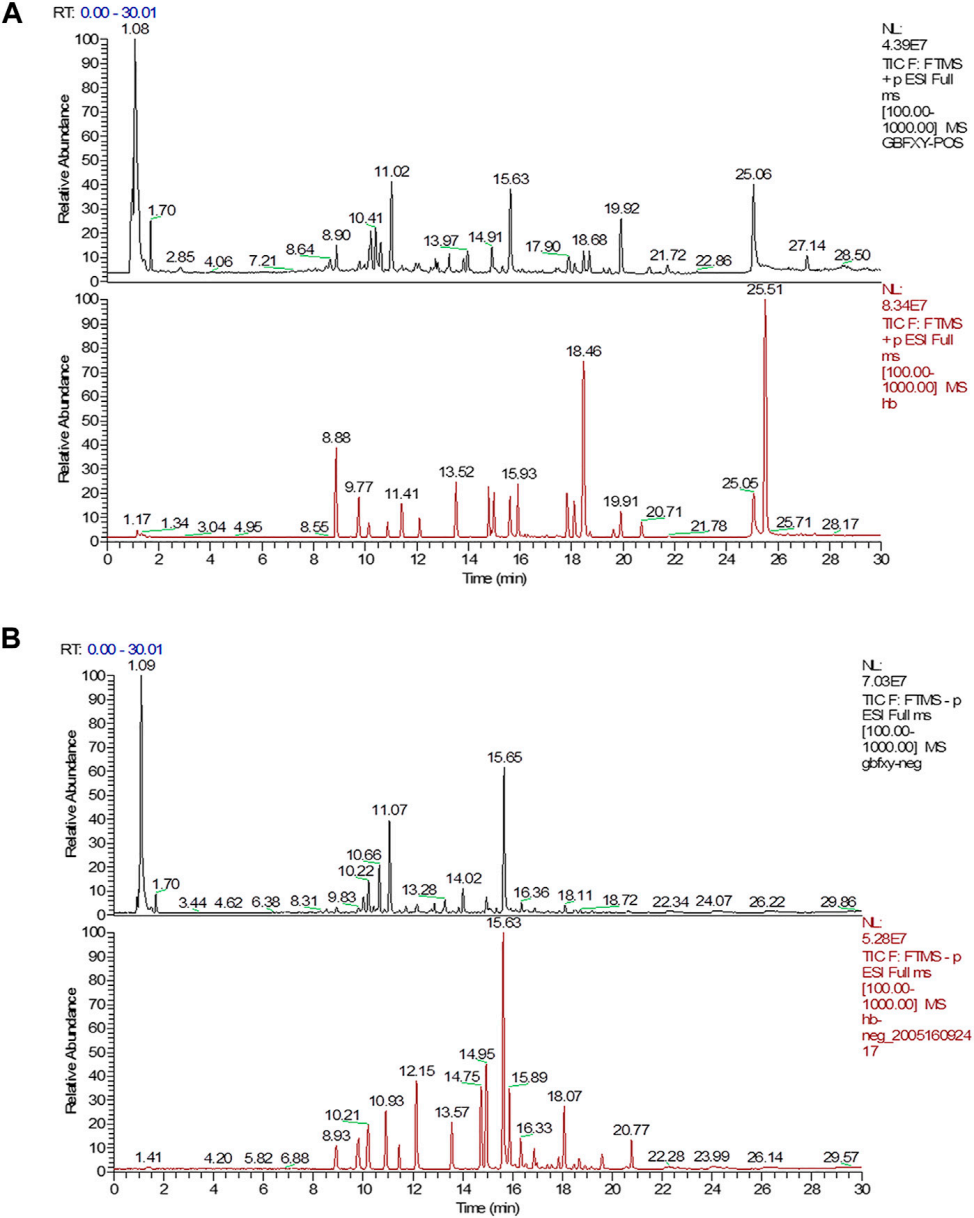
UPLC chromatograms analysis of GBFXD with the representative active ingredients. **(A)** The total chromatograms in positive ion mode (black, GBFXD; Red, standards); **(B)** The total chromatograms in negative ion mode (black, GBFXD; Red, standards).

The quality control of herbs was performed based on previous established methods (Dong et al., 2020) with minor modifications. Briefly, a Waters AQUITY H-Class ultra-high-performance liquid chromatograph (UPLC) was used and chromatographic separation was achieved by the AQUITY UPLC HSS T3 chromatographic column (2.1 × 100 mm, 1.8 μm). The column oven temperature was set at 25°C. The 0.1% formic acid in water (A) and 0.1% formic acid in acetonitrile (B) were chosen as the mobile phases at a flow rate of 0.2 ml/min. The elution conditions were as follows: 0 min: 10% B, 3 min: 10% B, 26 min: 90% B, 30 min: 10% B. The LTQ ORBITRAP XL tandem mass spectrometer was used with the following parameters: ion source: HESI, scan ranges: 100–1,000 m/z, and collision energy: 35. The positive ionization mode was used with the following parameters: heater temperature at 300°C, sheath gas flow rate at 45 arb, aux gas flow rate at 10 arb, spray voltage at 4 kV, capillary temperature at 300°C, capillary voltage at 35 V, and tube lens voltage at 110 V. In addition, the negative ionization mode was used the following parameters: heater temperature at 300°C, sheath gas flow rate at 45 arb, aux gas flow rate at 10 arb, spray voltage at 4 kV, capillary temperature at 300°C, capillary voltage at −35 V, tube lens voltage at −110 V. The reference standards included Prim-o-glucosylcimifugin (catalog number: 141021), Liquiritin (catalog number: 141029), Lobetyolin (catalog number: 140909), Astragaloside IV (catalog number: 140321), Magnolin (catalog number: 140,820), Schisandrol A (catalog number: 141211), Schisandrin A (catalog number: 19070202) (Chengdu Pufei De Biotech Co. Ltd., China), Calycosin-7-O-glucoside (catalog number: 120819), Isoliquiritin (catalog number: 120903), Glycyrrhizic acid (catalog number: 121213), Astragaloside I (catalog number: 120525), Astragaloside II (catalog number: 120528), Astragaloside III (catalog number: 120329), and Calycosin (catalog number: 120615) (Chengdu Herbpurity Co. Ltd., China). The detailed chemical information of GBFXD was shown in Figure 1 and Table 2.

**TABLE 2:** The chemical components identified from GBFXD.

No	Components	Formula	Adduct	MW (Da)	Standard RT (min)	GBFXD RT (min)	GBFXD m/z	ppm
1	Prim-o-glucosylcimifugin	C_22_H_28_O_11_	[M + H]^+^	469.17044	8.88	8.90	469.17053	0.196
			[M + FA-h]^−^	513.16027	8.93	8.93	513.16083	1.097
2	Calycosin-7-O-glucoside	C_22_H_22_O_10_	[M + H]^+^	447.12857	9.77	9.79	447.12833	−0.544
			[M + FA-h]^−^	491.11840	9.83	9.83	491.11917	1.563
3	Liquiritin	C_21_H_22_O_9_	[M-H]^−^	417.11801	10.21	10.22	417.11838	0.89
4	Lobetyolin	C_20_H_28_O_8_	[M + Na]^+^	419.16764	11.43	11.43	419.16721	−1.023
			[M + FA-h]^−^	441.17552	11.45	11.48	441.17624	1.624
5	Isoliquiritin	C_21_H_22_O_9_	[M-H]^−^	417.11801	12.15	12.18	417.1185	1.178
6	Astragaloside II	C_41_H_66_O_15_	[M + H]^+^	799.44745	12.53	12.53	799.44739	−0.072
7	Calycosin	C_16_H_12_O_5_	[M + H]^+^	285.07575	13.52	13.54	285.07587	0.421
			[M-H]^−^	283.06010	13.57	13.6	283.06116	3.745
8	Astragaloside IV	C_41_H_68_O_14_	[M + FA-h]^−^	829.45801	14.75	14.79	829.45795	−0.075
9	Astragaloside III	C_41_H_68_O_14_	[M + FA-h]^−^	829.458,012	14.95	14.99	829.45844	0.516
10	Glycyrrhizic acid	C_42_H_62_O_16_	[M + H]^+^	823.41106	15.62	15.63	823.41223	1.418
11	Magnolin	C_23_H_28_O_7_	[M + H]^+^	417.19078	17.83	17.83	417.19040	−0.91
12	Astragaloside I	C_45_H_72_O_16_	[M + Na]^+^	891.47126	18.11	18.14	891.47186	0.676
			[M + FA-h]^−^	913.47914	18.07	18.11	913.47974	0.655
13	Schisandrol A	C_24_H_32_O_7_	[M + Na]^+^	455.20402	18.46	18.47	455.20364	−0.845
14	Schisandrin A	C_24_H_32_O_6_	[M + H]^+^	417.22717	25.51	25.51	417.22668	−1.163

### Animal Experiment and Sample Preparation

**FIGURE 2:**
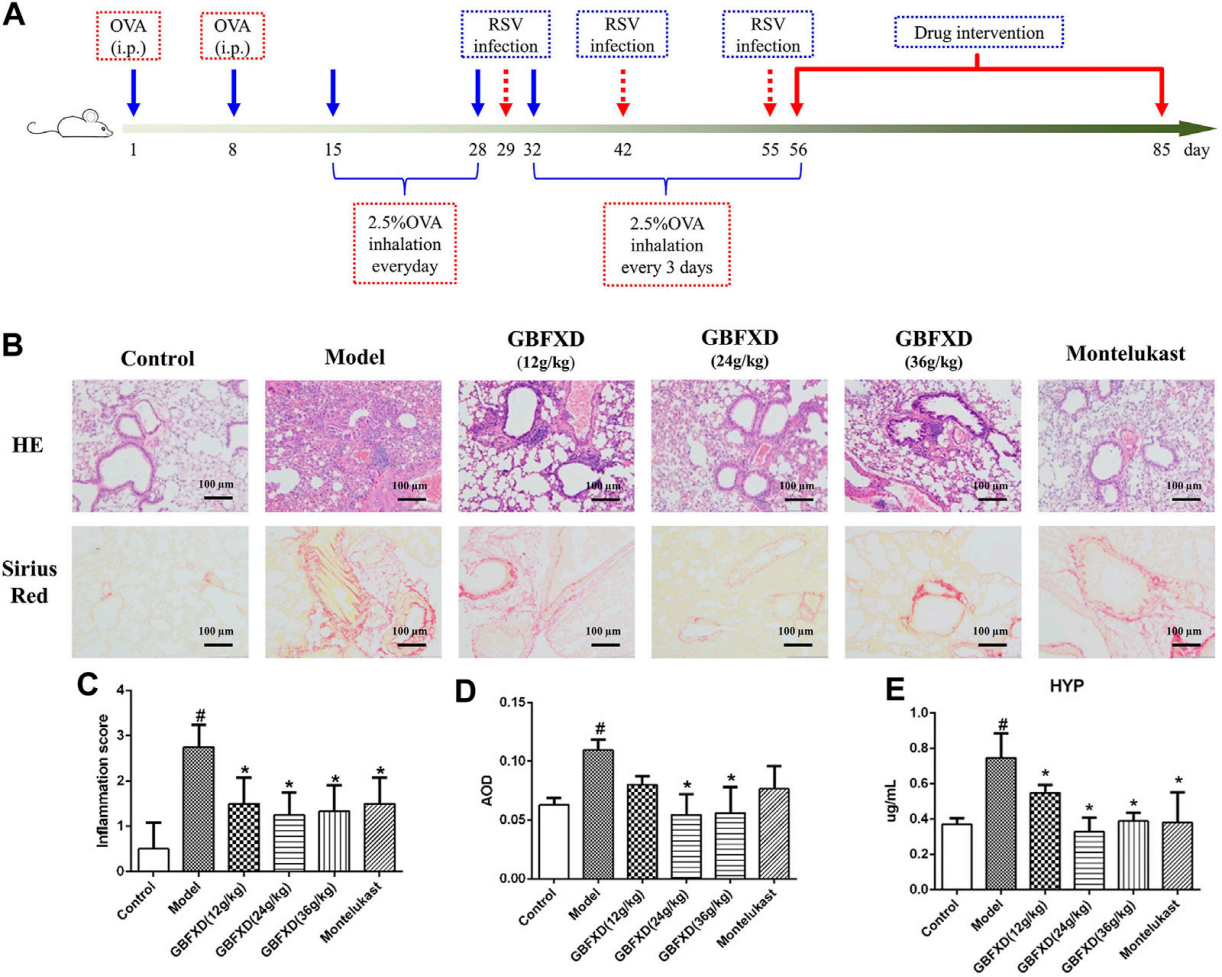
Effect of GBFXD on airway inflammation by hematoxylin and eosin (HE) staining, remodeling by Sirius red staining and HYP levels. **(A)** Experimental protocol to establish the murine OVA-RSV induced CRA murine model and the treatment strategy. **(B)** HE staining examined inflammatory infiltration and Sirius red staining identified collagen deposition (×200), Scale bar: 100 μm. **(C–D)** Total inflammation scores and the quantification of Sirius red staining by AOD in each group mice. **(E)** The HYP levels in lung of each group mice measured by ELISA. Values are presented as mean ± SD (*n* = 3 or 4 mice per group). #*p* < 0.05 compared to control group, and *p < 0.05 compared to model group.

Four-week-old female specific-pathogen-free BALB/c mice (weight: 16–18 g, 8 mice per group) were purchased from Charles River Laboratories (Beijing, China) and the murine model of CRA was established as previously described (Dong et al., 2020). Briefly, the mice were subjected to adaptive feeding for one week prior to treatment. On Days 1 and 8, mice were sensitized with an intraperitoneal injection of 0.2 ml normal saline (NS) containing 100 μg OVA mixed with 1 mg Al(OH)_3_. The mice were challenged with 2.5% erosolized OVA for 30 min/day from Days 15–28, and once every 3 days from Days 32–55. On Days 29, 42, and 55, the mice were intranasally administered with 50 μL (1.0 × 10^5^ TCID_50_/mL) respiratory syncytial virus (RSV). From Days 56–85, the mice in the treatment groups were intragastrically administered with different doses of GBFXD (12, 24, 36 g/kg) or Montelukast (2.6 mg/kg) per day. Montelukast, the leukotriene receptor antagonist and clinically most widely used for asthma remission (Lazarinis et al., 2018), was used as a positive control drug for GBFXD. In addition, the dosage conversion from human to mouse was calculated with reference to the book of “Research Methods in Pharmacology of Chinese Materia Medica” edited by Chen Qi (Chen, 2011). The dosage of GBFXD (36 g/kg) for mouse was converted from the clinical daily dosage for human. Due to the further increase in the concentration will enhance the difficulty of intragastric gavage, so the doses (12, 24, and 36 g/kg) are set and also consistent with our previous study (Huang et al., 2016; Lu et al., 2016; Dong et al., 2020). Mice in the control and model group received the same volume of NS. The procedure is shown in Figure 2A. All experimental procedures were performed and approved by the animal ethics committee of Nanjing University of Chinese medicine (No. 201809A019, No. 201810A021).

### Histopathology and Immunohistochemistry

The middle sections of the right lungs in each group were fixed with 4% paraformaldehyde. The samples were embedded in paraffin, cut into sections of 3–4 μm in thickness, and stained with hematoxylin-eosin (HE) or Sirius red. The inflammation score was determined as previously described (Xing et al., 2019). Other sections from each group were analyzed by immunohistochemistry to explore the expressions of E-cadherin, Vimentin (Servicebio, Wuhan, China), and α-SMA (Proteintech, Wuhan, China). The images were captured by a fluorescence microscope (Olympus BX43, Tokyo, Japan) at ×200 magnification. The results of staining and immunohistochemistry were analyzed by Image-Pro Plus 6.0 and quantified as average optical density (AOD).

### Enzyme-Linked Immunosorbent Assay

Hydroxyproline (HYP) levels in the lungs from each group were determined by ELISA (Westang, Shanghai, China), according to manufacturer’s protocol.

### Proteomics

#### Protein Extraction and Digestion

The frozen lung tissues were powdered in liquid nitrogen and proteins were extracted by the RIPA buffer (Beyotime, Haimen, China) with 10 mM dithiothreitol and protease inhibitor cocktails (Roche, Basel, Switzerland). The mixture was sonicated and centrifuged at 4°C and 30,000× *g* for 15 min. The supernatant was added to five volumes of cold acetone containing 10% (v/v) trichloroacetic acid, well mixed, and incubated at −20°C overnight. Afterward, the mixture was centrifuged again at 4°C, 30,000× *g* and the suspension was discarded. The sediment was washed three times with cold acetone, air-dried, and dissolved in RIPA buffer. The extracted proteins were quantified by a BCA kit (Thermo Fisher Scientific, United States); 300 µg proteins from each sample were mixed with sequencing-grade trypsin (Promega, Madison, WI, United States) at an enzyme to protein ratio of 1:50 and incubated for 16 h at 37°C. The peptides acquired from enzymatic digestion were dried by vacuum centrifugation.

#### iTRAQ Labeling and High-pH Reversed-phase Fractionation

The obtained peptides were processed by 8-plex iTRAQ reagent (AB Sciex, Framingham, MA, United States) per manufacturer’s instructions. The control samples were labeled with 115 iTRAQ tags, the model samples were labeled with 116 iTRAQ tags, and the GBFXD samples were labeled with 113 iTRAQ tags. High pH RP fractionation was performed with the U3000 HPLC chromatography system (Thermo Fisher Scientific, United States). The iTRAQ-labeled peptides were reconstituted with 100 µL high pH RP buffer A (98% H_2_O, 2% acetonitrile, pH 10.0) and loaded onto a C18 column (250 × 3 mm, 5 µm particle size, Phenomenex, United States). The column was eluted (flow rate: 0.2 ml/min) with the following parameters: 3–18% buffer B (2% H_2_O, 98% acetonitrile, pH 10.0) for 30 min, 18–32% B for 15 min, 32–98% B for 6 min, and 98% B for 15 min. The elution was monitored by detecting the absorbance at 214 nm.

#### LC-MS/MS Analysis

The peptides were re-dissolved in buffer A (2% acetonitrile, 0.1% formate) and centrifuged (4°C, 20,000× *g*, 10 min). Using the Nano LC System auto sampler (Thermo Fisher Scientific, United States), 10 µL peptides were loaded onto a 2 cm C18 trap column and eluted onto a 15 cm analytical C18 column with an inner diameter of 75 µm using a 3–55% buffer B (84% acetonitrile, 0.1% formate). The elution process lasted 112 min at a flow rate of 300 nL/min. With 2.2 kV voltage, the peptides were ionized by nano-electrospray ionization and data-dependent MS/MS was performed by the LTQ-Orbitrap XL mass spectrometer (Thermo Fisher Scientific). With the MS1 full scan (resolution: 60,000), the five most abundant precursor ions above the 5,000 counts threshold were chosen for MS/MS analysis along with a dynamic exclusion duration of 60 s. Normalized collision energy for high-energy collision dissociation was set to 40.0 and then the ion fragments were tested in the Orbitrap at a resolution of 7,500 with scan ranges of 350–1800 Da (MS1) and 100–1800 Da (MS2).

#### Identification and Quantification of Proteins

Raw data was analyzed by Proteome Discoverer (version: 2.0, Thermo Fisher Scientific, United States). The SEQUEST search engine was used to identify the proteins, and a mass tolerance of 10 ppm or 0.02 Da was permitted for intact peptides or fragmented ions with an allowance of no more than two missed cleavages with trypsin digestion. Except for iTRAQ labels, the fixed modification was carbamidomethylation (C), whereas the variable modifications were oxidation (M) and deamidation (NQ). With SEQUEST probability analysis, peptides at the 99% confidence interval were counted as identified, which may reduce the probability of false identifications. At least two unique peptides were required to quantify the proteins. To weigh and normalize the quantitative protein ratios, the median ratio in SEQUEST was applied. The DEPs were identified by fold change >1.5 or <0.67 with FDR < 1%.

#### Bioinformatic Analysis of DEPs

OmicsBean (http://www.omicsbean.cn), including gene ontology (GO) enrichment and Kyoto Encyclopedia of Genes and Genomes (KEGG) pathway analysis, was used to analyze DEPs. In additon, the Heat map was produced by MetaboAnalyst 5.0 (https://www.metaboanalyst.ca).

### Real-Time PCR

Total RNA in the lung was extracted by the Fast Pure Cell/Tissue Total RNA Isolation Kit (Vazyme Biotech Co., Ltd., Nanjing, China), from which cDNA was synthesized with the HiScript® II Q RT SuperMix for qPCR (Vazyme Biotech Co., Ltd., Nanjing, China) and quantified using the ChamQ universal SYBR qPCR Master Mix (Vazyme Biotech Co., Ltd., Nanjing, China). The results were analyzed using the QuantStudio 7 Flex detection system (Applied Biosystems Co., United States) and the 2^−ΔΔCT^ method to assess the levels of mRNAs encoding targeted genes normalized to *GAPDH*. The primers are shown in Table 3.

**TABLE 3:** Primer sequences.

Gene	Forward primer	Reverse primer
*Col1a1*	GAC​AGG​CGA​ACA​AGG​TGA​CAG​AG	CAG​GAG​AAC​CAG​GAG​AAC​CAG​GAG
*Col1a2*	GGC​AAC​AGC​AGG​TTC​ACC​TAC​TC	GTC​AGC​ACC​ACC​AAT​GTC​CAG​AG
*Col4a1*	ATG​GCA​TTG​TGG​AGT​GTC​AAC​CTG	CTT​CAC​CTG​TCA​AAC​CTG​GCT​GTC
*Col4a2*	AGC​CTG​GTG​TAC​TCG​GTC​TTC​C	GGT​CGC​CTT​TGG​GTC​CTT​TGG
*LAMA3*	GTC​CTA​GCC​AAT​TCC​GTG​TCC​TTG	TGC​TCT​CCA​GAT​TGA​CTT​GCG​ATG
*LAMA5*	CGC​CAG​CAA​GGT​CAA​GGT​GTC	GCA​GCA​AGG​TCG​GCA​AGG​TC
*LAMB2*	TGG​CTG​TCT​ACC​TGG​CAT​CTG​G	TGG​GTC​CCG​CAT​GTC​CTT​GG
*LAMB3*	GCC​GAG​GAA​GCA​GCA​TCA​AGG	CCT​GTT​GGA​TGA​GAA​GCC​GTG​TG
*Fn1*	CTA​TAG​GAT​TGG​AGA​CAC​GTG​G	CTG​AAG​CAC​TTT​GTA​GAG​CAT​G
*GAPDH*	GTG​GAG​TCA​TAC​TGG​AAC​ATG​TAG	AAT​GGT​GAA​GGT​CGG​TGT​G

### Western Blotting

The proteins were extracted from the lungs in each group and measured by the BCA kit. Equal amounts of proteins were separated by SDS-PAGE, transferred to polyvinylidene difluoride membranes (0.45 μm, Millipore, United States), and incubated with primary antibodies including AKT (1:2,000 dilution), P-AKT (1:5,000 dilution), RTN4 (1:500 dilution) (Proteintech, Wuhan, China), Col1a2 (1:1,000 diluted), Col4a1 (1:1,000 dilution), PI3K (1:1,000 dilution) (Immunoway, Plano, TX, United States), P-PI3K (1:1,000 dilution), GAPDH (1:10,000 dilution (Affinity Biosciences, Cincinnati, OH, United States), PTEN (1:1,000 dilution) (Cell Signaling Technology Inc., United States) and Vimentin (1:1,000 dilution) (Arigo, Shanghai, China). The ChemiDoc™ MP Imaging system (Bio-Rad Co., United States) was used to quantify the protein bands with the Image Lab software (Bio-Rad Co., United States). The values were expressed as intensities normalized to GAPDH or their respective total proteins.

### Statistical Analysis

Data were expressed as mean and standard deviation (SD). One-way analysis of variance (ANOVA) followed by the Dunnett’s *post hoc* test was used to determine statistical significances between groups. Differences with *p* < 0.05 were considered significant. All statistical analyses were performed in GraphPad Prism 6.0 (GraphPad Software Inc., San Diego, CA, United States).

## Results

### Identification of GBFXD Components

A total of 14 chemical compounds were identified in GBFXD, namely Prim-o-glucosylcimifugin, Calycosin-7-O-glucoside, Liquiritin, Lobetyolin, Isoliquiritin, Astragaloside II, Calycosin, Astragaloside IV, Astragaloside III, Glycyrrhizic acid, Magnolin, Astragaloside I, Schisandrol A, and Schisandrin A (Figures 1A,B and Table 2).

### GBFXD Ameliorated Airway Inflammation and Remodeling in a Murine Model of CRA

**FIGURE 3:**
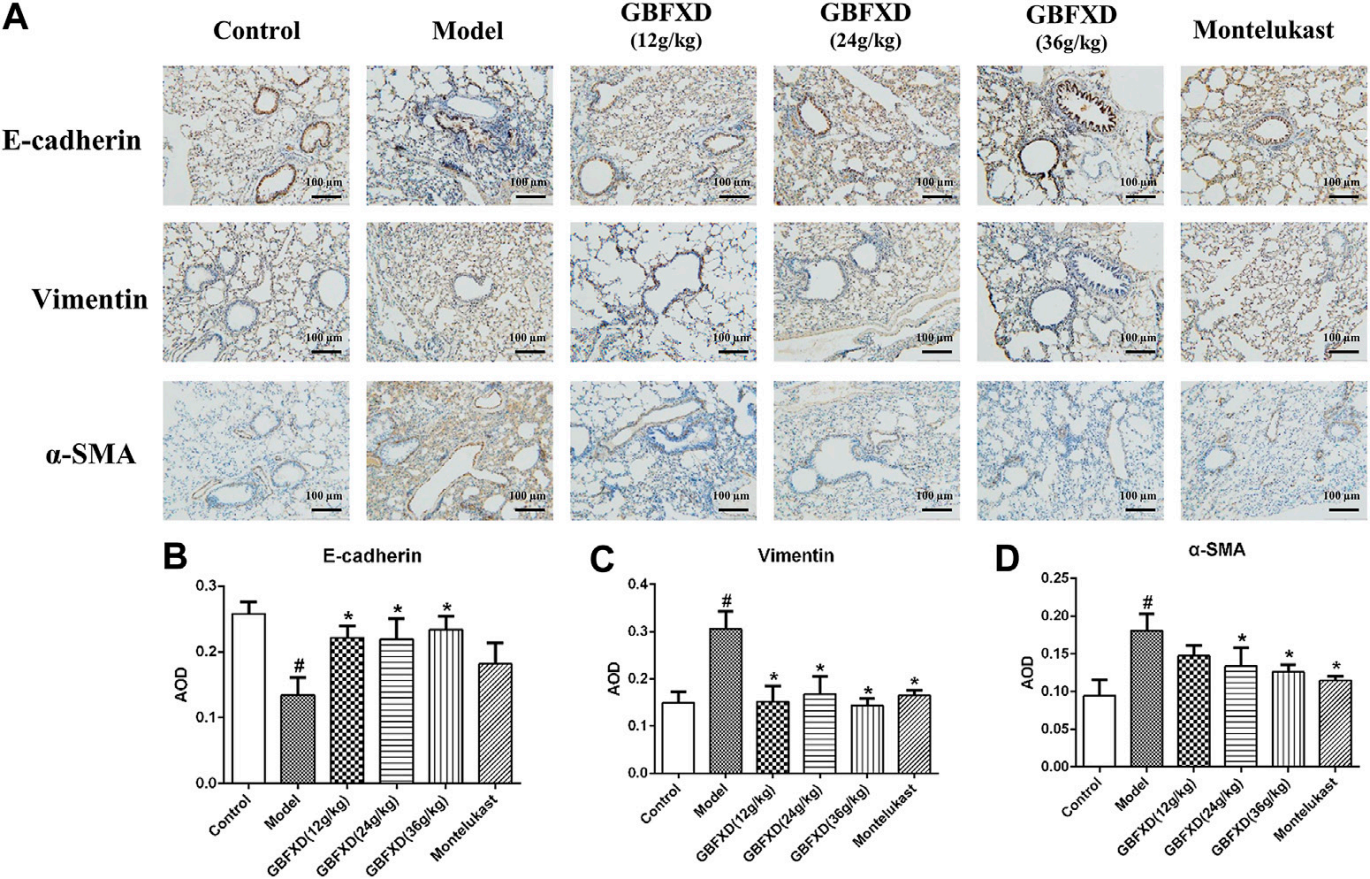
GBFXD decreased epithelial-to-mesenchymal transition in CRA mice. **(A)** Markers of lung EMT were performed by immunohistochemical staining (×200), Scale bar: 100 μm. **(B–D)** Relative protein expressions of E-cadherin (epithelial mark), Vimentin and α-SMA (mesenchymal marks) were quantified by AOD. Values are presented as mean ± SD (*n* = 3 mice per group). #*p* < 0.05 compared to control group, and *p < 0.05 compared to model group.

HE and Sirius red staining were analyzed to determine airway inflammation and remodeling. The mice in the model group exhibited significantly increased inflammation and collagen deposition in the respiratory tract (Figures 2B–D). These pulmonary pathological symptoms were obviously alleviated in the mice with GBFXD treatment (24 g/kg or 36 g/kg) compared with those in the model group, although the administration of GBFXD (12 g/kg) and Montelukast did not improve collagen deposition (Figures 2B,D). For molecules relevant to airway remodeling, the expressions of Vimentin, α-SMA and HYP were increased, while the E-cadherin was reduced in CRA mice. More importantly, GBFXD (24 g/kg or 36 g/kg) drastically restored the expressions of those proteins (Figures 2E, 3A–D). In addition, the changes in Vimentin were further confirmed by Western blotting (Supplementary Figure S1). The above results confirmed that GBFXD could relieve airway inflammation and remodeling in the murine model of CRA.

### DEPs Identification by iTRAQ-Based Proteomics

**FIGURE 4:**
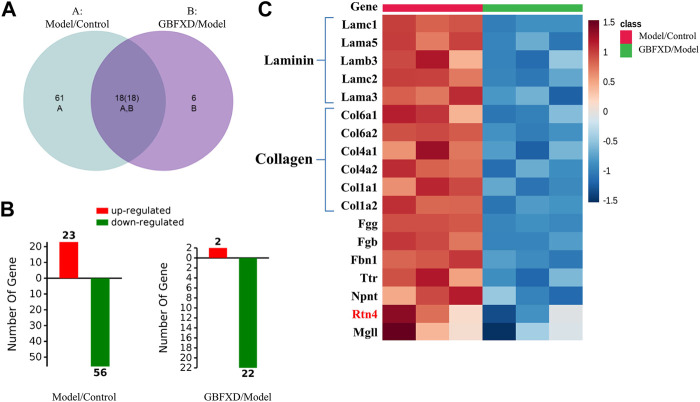
The differentially expressed proteins (DEPs) in lung between Model/Control and GBFXD/Model. **(A)** Venn-diagram of DAPs. **(B)** The left was DAPs involved in Model/Control and the right was GBFXD/Model (the numbers of upregulated, downregulated proteins characterized by Red and Green, respectively). **(C)** Heat map of overlap DEPs.

To explore the underlying mechanisms of GBFXD in CRA, iTRAQ-based proteomics was used to identify the DEPs in the control, model and GBFXD (36 g/kg) groups. In addition, the dose of GBFXD is the same as before (Liu et al., 2019; Xing et al., 2019). A total of 2,319 proteins were identified, among which 1,472 were quantified. As shown in Figures 4A,B 79 DEPs in the model/control were identified (Table 4, upregulated: 23, downregulated: 56), whereas 24 DEPs were identified in the GBFXD/model group (Table 5, upregulated: 2, downregulated: 22). There were 18 overlapping DEPs, which were upregulated in the model/control group, and downregulated by GBXFD treatment (Figure 4C). Further analysis found that these proteins were mostly collagen and laminin, which were extracellular matrix (ECM) proteins and closely related to airway remodeling. Downregulated expression of fibronectin (Fn1), a key marker of airway remodeling, was also observed after GBFXD treatment.

**TABLE 4:** The DAPs of lung in the model group compared with the control group.

Uniprot ID	Fold change	Gene name	Protein name
B2RQQ1	0.60	MYH6	Myosin, heavy polypeptide 6
B2RWW8	0.28	MYH8	Myosin, heavy polypeptide 8
P13542	0.30	Myh8	Myosin-8
B2RWX0	0.31	MYH1	Myosin, heavy polypeptide 1
P68134	0.60	Acta1	Actin, alpha
B2RXX9	0.59	MYH7	Myosin, heavy polypeptide 7, beta
G3UW82	0.36	Myh2	Myosin, heavy polypeptide 2
Q5SX39	0.27	Myh4	Myosin-4
P13541	0.22	Myh3	Myosin-3
P58771	0.43	Tpm1	Tropomyosin alpha-1 chain
Q564G1	0.57	TPM1	Tropomyosin 1, alpha
A0A2R2Y2P8	0.52	TPM1KAPPA	Tropomyosin 1 kappa
Q9JI91	0.40	Actn2	Alpha-actinin-2
P07310	0.33	Ckm	Creatine kinase M-type
B1AR69	0.49	Myh13	Myosin, heavy polypeptide 13
P08730	0.46	Krt13	Keratin, type I cytoskeletal 13
F8VQJ3	1.57	Lamc1	Laminin subunit gamma-1
P09541	0.55	Myl4	Myosin light chain 4
Q9WUB3	0.53	Pygm	Glycogen phosphorylase
Q61001	1.66	*Lama*5	Laminin subunit alpha-5
Q545G1	0.26	MYLPF	—
P09542	0.22	Myl3	Myosin light chain 3
Q3TGR2	1.61	FGB	Fibrinogen C-terminal domain-containing protein
Q8R429	0.35	Atp2a1	Sarcoplasmic/endoplasmic reticulum calcium ATPase 1
P20801	0.44	Tnnc2	Troponin C
P07744	0.49	Krt4	Keratin, type II cytoskeletal 4
Q3UER8	1.95	Fgg	Fibrinogen gamma chain
Q5SVI8	0.5	MYL7	Uncharacterized protein
J3QQ13	0.66	Tnnt2	Troponin T
P04117	0.54	Fabp4	Fatty acid-binding protein
Q02788	1.66	Col6a2	Collagen alpha-2(VI) chain
Q04857	1.55	Col6a1	Collagen alpha-1(VI) chain
Q91V88	3.54	Npnt	Nephronectin
P02463	2.46	Col4a1	Collagen alpha-1(IV) chain
P21550	0.34	Eno3	Beta-enolase
Q61554	4.09	Fbn1	Fibrillin-1
Q01149	4.06	Col1a2	Collagen alpha-2(I) chain
Q61087	1.79	Lamb3	Laminin subunit beta-3
P16015	0.50	Ca3	Carbonic anhydrase 3
O88990	0.63	Actn3	Alpha-actinin-3
Q61789	1.57	Lama3	Laminin subunit alpha-3
Q7TQ48	0.54	Srl	Sarcalumenin
Q545T7	0.45	MYL1	Uncharacterized protein
P11087	4.66	Col1a1	Collagen alpha-1(I) chain
G5E874	2.52	Lamc2	Laminin subunit gamma-2
B2RQQ8	2.22	COL4A2	Collagen, type IV, alpha 2
P19123	0.65	Tnnc1	Troponin C
P03958	0.50	Ada	Adenosine deaminase
P07309	1.83	Ttr	Transthyretin
Q99P72	1.77	Rtn4	Reticulon-4
Q8BMF4	0.66	Dlat	Dihydrolipoyllysine-residue acetyltransferase component of pyruvate dehydrogenase complex
Q8R1M2	0.64	H2afj	Histone H2A.J
P04247	0.35	Mb	Myoglobin
P32848	0.39	Pvalb	Parvalbumin alpha
B7ZN13	0.50	ALDH3A1	Aldehyde dehydrogenase
Q3TSV1	0.61	DCN	Decorin
E9QK82	0.52	Mpz	Myelin protein P0
D3YYS6	1.65	Mgll	Monoglyceride lipase
I6L985	1.68	IGH	Igh protein
D3YU50	0.28	Mybpc1	Myosin-binding protein C
Q8VHX6	0.58	Flnc	Filamin-C
A0A0R4J1B0	0.44	Tnnt3	Troponin T
Q3U254	2.24	EMILIN1	Uncharacterized protein
O55124	0.62	MYOM2	M-protein
P05125	0.43	Nppa	Natriuretic peptides A
A2AQA9	0.38	Neb	Nebulin
Q9QZS0	3.65	Col4a3	Collagen alpha-3(IV) chain
Q9R0Y5	0.65	Ak1	Adenylate kinase isoenzyme 1
Q99MQ4	0.63	Aspn	Asporin
G3X8U3	1.92	2210016F16Rik	Queuosine salvage protein
P04370	0.57	Mbp	Myelin basic protein
Q08189	0.53	Tgm3	Protein-glutamine gamma-glutamyltransferase E
Q9D881	0.65	Cox5b	Cytochrome c oxidase subunit 5 B
Z4YJF5	0.46	Myom1	Myomesin-1
Q3TLQ9	1.59	EIF4B	RRM domain-containing protein
Q9JKK7	0.62	Tmod2	Tropomodulin-2

**TABLE 5:** The DAPs of lung in the GBFXD group compared with the model group.

Uniprot ID	Fold change	Gene name	Protein name
Q61292	0.53	Lamb2	Laminin subunit beta-2
F8VQJ3	0.51	Lamc1	Laminin subunit gamma-1
Q61001	0.53	Lama5	Laminin subunit alpha-5
Q3TGR2	0.55	FGB	Fibrinogen C-terminal domain-containing protein
P02535	0.66	Krt10	Keratin, type I cytoskeletal 10
Q3TG75	1.63	OAT	—
Q3UER8	0.45	Fgg	Fibrinogen gamma chain
E9PV24	0.61	Fga	Fibrinogen alpha chain
P11276	0.62	Fn1	Fibronectin
Q02788	0.56	Col6a2	Collagen alpha-2(VI) chain
Q04857	0.61	Col6a1	Collagen alpha-1(VI) chain
Q91V88	0.26	Npnt	Nephronectin
P02463	0.44	Col4a1	Collagen alpha-1(IV) chain
Q61554	0.20	Fbn1	Fibrillin-1
Q01149	0.25	Col1a2	Collagen alpha-2(I) chain
Q61087	0.54	Lamb3	Laminin subunit beta-3
Q61789	0.63	Lama3	Laminin subunit alpha-3
P11087	0.21	Col1a1	Collagen alpha-1(I) chain
G5E874	0.35	Lamc2	Laminin subunit gamma-2
B2RQQ8	0.42	COL4A2	Collagen, type IV, alpha 2
P07309	0.44	Ttr	Transthyretin
Q99P72	0.56	Rtn4	Reticulon-4
D3YYS6	0.66	Mgll	Monoglyceride lipase
P54823	1.76	Ddx6	Probable ATP-dependent RNA helicase DDX6

### Bioinformatic Analysis of DEPs

**FIGURE 5:**
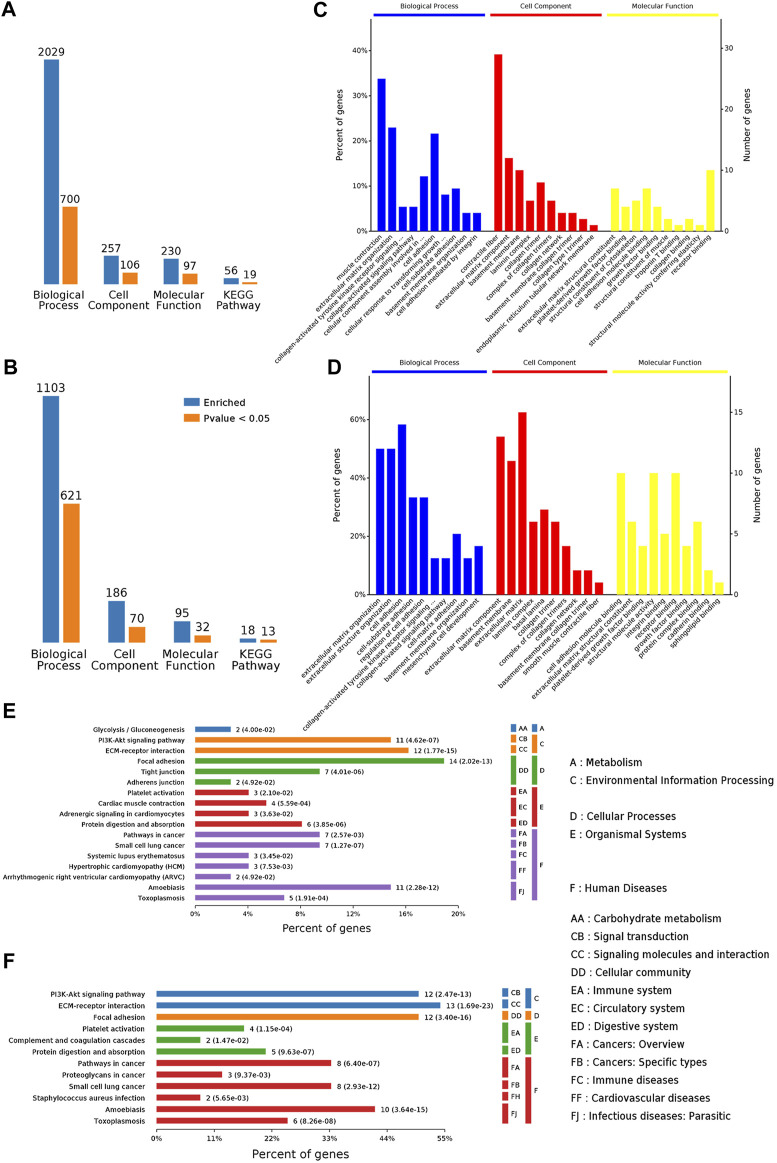
Functional annotation and categories of DAPs. **(A–B)** Bioinformatics analysis of DEPs by categories: BP, CC, MF, and KEGG pathway, respectively (A: Model/Control, B: GBFXD/Model). Counts for each category represent the total associated terms in the database with the query protein list. Terms are statistically significant with a *p*-value <0.05. **(C–D)** The ten most significantly enriched terms of the GO hierarchy, information of percentage, and number of involved proteins in a term are shown in the left and right *y*-axes, respectively (C: Model/Control, D: GBFXD/Model). **(E–F)** Enriched KEGG pathways of DAPs (E: Model/Control, F: GBFXD/Model). The number of involved proteins and corresponding *p*-values are shown on the right side of the column.

GO annotations including biological process (BP), cellular component (CC), and molecular function (MF) were used to obtain the functional information of DEPs. As shown in Figures 5A,B, the DEPs of the Model/control group indicated significant enrichment of 700 BP terms, 106 CC terms, and 97 MF terms. After GBFXD treatment, the DEPs of the GBFXD/Model group showed significant enrichment of 621 BP terms, 70 CC terms, and 32 MF terms. The top 10 significantly enriched GO terms were shown in Figures 5C,D.

The top three terms of DEPs for BP from the model/control group were muscle contraction, ECM organization, and cell adhesion. Most proteins were involved in contractile fiber, ECM component, collagen trimer for CC, and receptor binding, ECM structural constituent, cell adhesion molecule binding for MF (Figure 5C). The most enriched BP terms of DEPs for BP from the GBFXD/model group include cell adhesion, ECM organization, and extracellular structure organization (Figure 5D). In addition, the ECM, ECM component, basement membrane for CC and receptor binding, cell adhesion molecule binding, structural molecule activity for MF were the most enriched. Consequently, the GO analysis indicated that GBFXD may impact ECM related to airway remodeling.

According to KEGG pathway enrichment analysis, the DEPs from the model/control and GBFXD/model groups were shown to be simultaneously involved in ECM-receptor interactions, the PI3K/AKT signaling pathway, and focal adhesion, which were the top three significantly enriched pathways (Figures 5E,F) and have been reported to participate in airway remodeling in asthma (Athari, 2019; Yap et al., 2019; Zhou et al., 2019; Ali et al., 2020).

### Validation of DEPs

**FIGURE 6:**
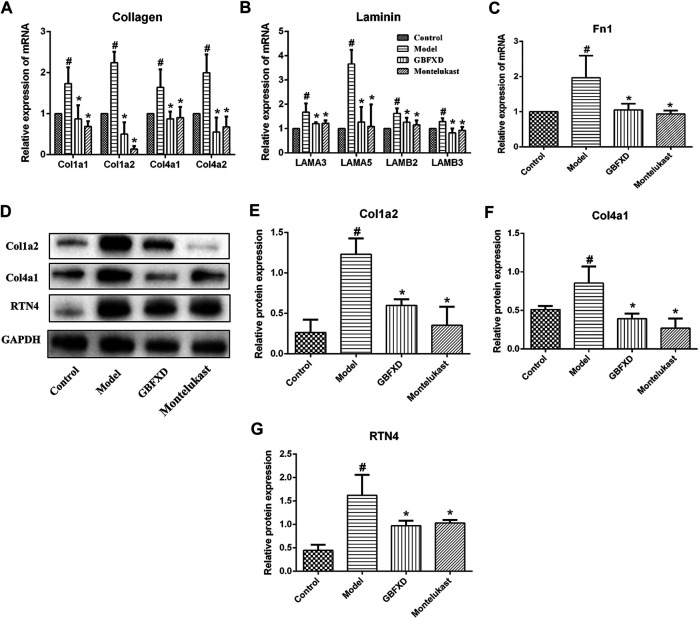
Validation of DEPs identified in proteomics. **(A–C)** The mRNA levels of Collagen (Col1a1, Col1a2, Col4a1, Col4a2), Laminin (LAMA3, LAMA5, LAMB2, LAMB3) and Fn1 in lung were measured by Real-time PCR. **(D)** The proteins of Col1a2, Col4a1, and RTN4 were analyzed by Western Blot. **(E–G)** Quantification of Col1a2, Col4a1, and RTN4. Values are presented as mean ± SD (*n* = 3 or 4 mice per group). #*p* < 0.05 compared to control group, and *p < 0.05 compared to model group.

The expression of mRNAs encoding collagen (*Cola1*, *Col1a2*, *Col4a1*, *Col4a2*), laminin (*LAMA3*, *LAMA5*, *LAMB2*, *LAMB3*), and *Fn1* in the lungs of the model group were significantly higher than those in the control group (Figures 6A–C). Meanwhile, the expression levels of these mRNAs were decreased in mice treated with GBFXD or Montelukast. Data on the protein levels of Col1a2, Col4a1, and RTN4 also showed consistent results (Figures 6D–G) with iTRAQ proteomic analysis and the trends in the mRNA levels.

### GBFXD Probably Inhibited the Activation of the PI3K/AKT Signaling Pathway Mediated by RTN4

**FIGURE 7:**
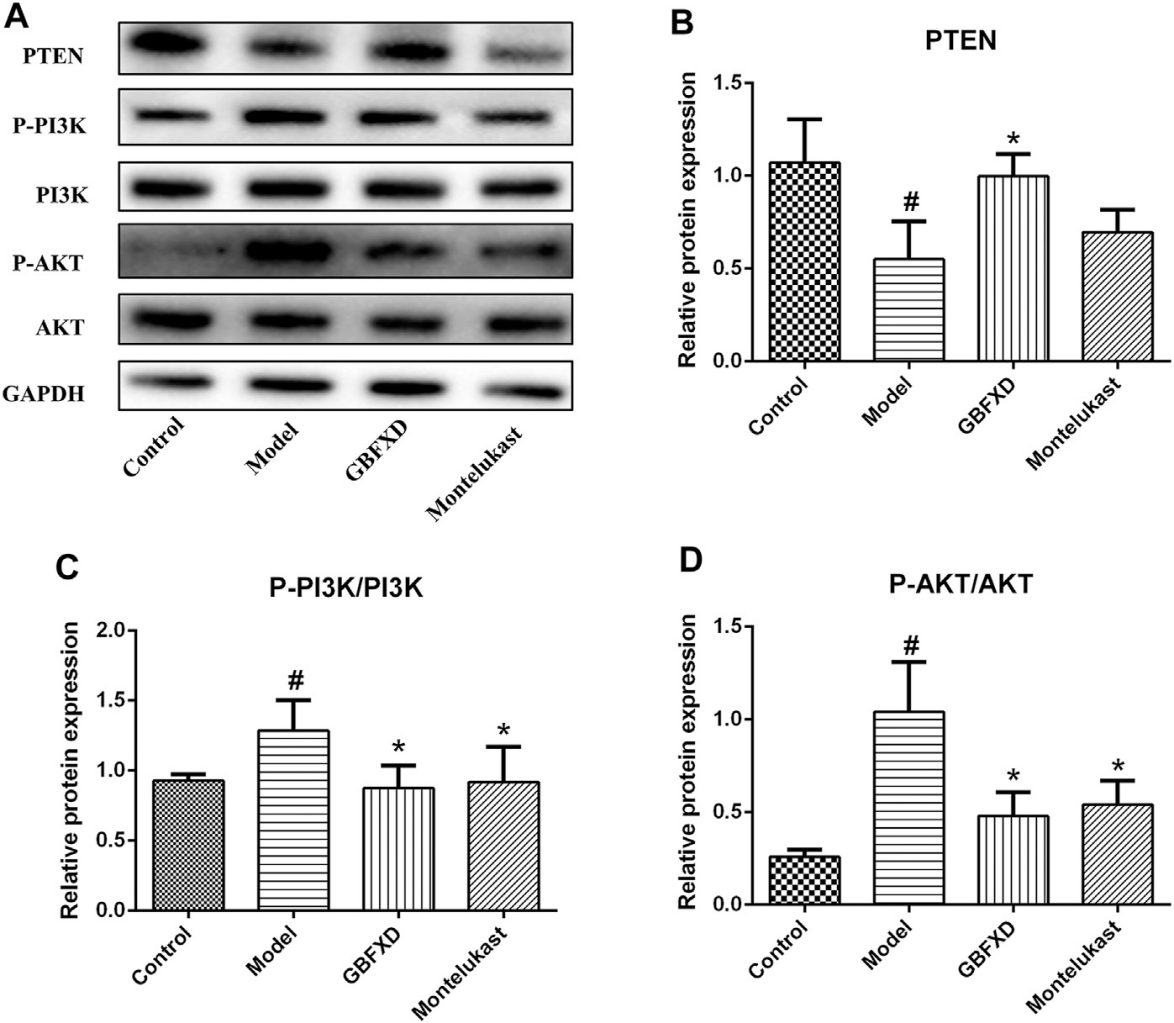
Effect of GBFXD on PI3K/AKT signaling pathway. **(A)** Western blotting was used to detect the protein expressions of PTEN, P-PI3K, PI3K, P-AKT, and AKT. **(B–D)** Quantification of PTEN, P-PI3K/PI3K and P-AKT/AKT. Values are presented as mean ± SD (*n* = 3 or 4 mice per group). #*p* < 0.05 compared to control group, and *p < 0.05 compared to model group.

We further confirmed the pharmacological mechanisms of GBFXD. Recent publications suggested that the RTN4 promoted angiogenesis in proliferative diabetic retinopathy via PI3K/Akt signal pathway and epithelial-mesenchymal transition in non-small cell lung cancer cells (Zhang et al., 2017; Wu et al., 2018). Based on our proteomics results, GBFXD could regulate PI3K/AKT pathway and RTN4 was also an overlapping important DEP. In addition, our verification experiments also proved that GBFXD could regulate the expression of RTN4. Therefore, we hypothesized that GBFXD could alleviate airway remodeling by inhibiting the pathway of PI3K/AKT via RTN4. The expression of PTEN was lower, and the phosphorylation of PI3K and AKT were remarkably higher in the model group (Figures 7A–D) compared to the control group. More importantly, the dysregulation was reversed after GBFXD treatment. Consequently, the data indicated that GBFXD ameliorated airway remodeling by inhibiting the activation of the PI3K/AKT signaling pathway via RTN4 to reduce ECM deposition possibly.

## Discussion

Asthma is a common chronic inflammatory respiratory disease of highly prevalence worldwide. There is currently no treatment to cure this disease, but some patients may spontaneously enter asthma remission later in life (Carpaij et al., 2017). Asthma remission often refers to the state in which patients are no longer burdened by the disease to some extent, and no longer require any asthma medication. However, most of the patients might still have airway remodeling, hyperresponsiveness, or declined lung function, and very few can go into complete asthma remission with no respiratory symptoms (Broekema et al., 2011; Carpaij et al., 2017). Even though some therapies such as ICS or biological treatment can fully control the symptoms, they have not been associated with real remission. Recently, immunotherapy has been shown to have a vital effect on asthma remission and cumulating evidence indicates that TCM has unique advantages in treating asthma primarily by regulating immunity (Bao et al., 2019; Cui et al., 2019; Yao et al., 2019). GBFXD was shown to be effective in improving the life of asthmatic children and reducing the rate of relapses. Moreover, findings from our previous study have indicated that GBFXD could regulate Th1/Th2, macrophage polarization (Liu et al., 2019), CD4^+^/CD8^+^ (Lu et al., 2016) and Treg cells (Dong et al., 2020) to enhance the immune activity, which has triggered our increasing interest in further research on GBFXD.

Asshown in the results of the HE and Sirius red staining, airway inflammatory infiltration and collagen deposition were effectively relieved after GBFXD treatment. Airway remodeling is closely related to the dysregulation of epithelial-mesenchymal transition (EMT) by which epithelial cells transform into the mesenchymal phenotype after epithelial damage (Guan et al., 2020). EMT is mainly associated with decreased levels of epithelial phenotypic proteins including E-cadherin, ZO-1, and increased levels of mesenchymal phenotypic proteins such as N-cadherin, Vimentin, and α-SMA (Bartis et al., 2014). Subsequently, we showed the pharmacological effects of GBFXD on typical markers of EMT (Vimentin, α-SMA, and E-cadherin). HYP, an indicator of the collagen content in the lung tissue, is associated with the occurrence of lung fibrosis (Sun et al., 2020). In this study, HYP levels were significantly reduced in mice treated with GBFXD, which further confirmed the effect of GBFXD on airway remodeling.

However, the detailed mechanisms of GBFXD on alleviating airway remodeling in CRA remained unclear, which were further explored by proteomic analyses. The identified overlapping DEPs were mostly collagen and laminin. Reticular basement membrane with ECM protein deposition is a characteristic of airway remodeling. The ECM proteins include structural proteins, adhesion proteins, glycosaminoglycans, and proteoglycans, and examples include collagen, laminin, and Fn1 (Olczyk et al., 2014). More importantly, the deposition of ECM molecules is increased in asthmatic airways (Royce et al., 2012; Mostaço-Guidolin et al., 2019). In this study, increased mRNA or protein levels of DEPs were observed for collagen (Col1a1, Col1a2, Col4a1, Col4a2), laminin (LAMA3, LAMA5, LAMB2, LAMB3), and Fn1 in CRA mice, while the expressions of these molecules decreased after GBFXD treatment. Collectively, our validation of DEPs was consistent with proteomic findings.

The KEGG analysis showed that GBFXD could regulate pathways related to airway remodeling, such as ECM-receptor interactions, focal adhesion, and the PI3K/AKT signaling pathway, which were the top three significantly enriched pathways containing the most DEPs for both Model/Control and GBFXD/Model. RTN4 was an important common DEP and downregulated after GBFXD treatment. A previous study showed that RTN4 was primarily localized in the ER and was commonly known as Nogo-B in the peripheral tissues including the lung (Pathak et al., 2018). Accumulating research also reported the importance of RTN4 in tissue repairing and hepatic fibrosis (Yu et al., 2009; Tashiro et al., 2013). In addition, RTN4 is necessary for the migration and contraction of airway smooth muscle cells in airway remodeling in chronic asthma (Xu et al., 2011), promoting angiogenesis in proliferative diabetic retinopathy via the PI3K/Akt signal pathway (Zhang et al., 2017), and facilitating EMT in non-small cell lung cancer (Wu et al., 2018). However, the relationship of RTN4 and PI3K/AKT pathway with airway remodeling in CRA was still unclear. According to our present findings (Figures 6D–G, 7A–D), GBFXD regulated RTN4 and suppressed the activation of the PI3K/AKT pathway. Recent research has demonstrated an association between the PI3K/AKT pathway and airway remodeling (Hong et al., 2017; Shao et al., 2019) and the KEGG analysis of our proteomics also confirmed this finding. Consequently, we hypothesize that potential mechanisms of GBFXD on airway remodeling in CRA may be related to inhibiting RTN4 and subsequently suppressing the activation of the PI3K/AKT signaling pathway to reduce the deposition of ECM related molecules. More in-depth research will be performed to prove the conclusion. In addition, GBFXD in CPA mainly regulated the pathways of mitochondrial energy metabolism and macrophage polarization which were also related to airway inflammation and remodeling as reported in our previous study (Liu et al., 2019). Herein, there may be different pathological mechanisms between CPA and CRA, which may explain the differences in HE, PAS staining and Penh between CPA and CRA mice in previous studies (Liu et al., 2019) and warrant further exploration. The proteomics data have also confirmed that GBFXD could regulate airway remodeling in CPA or CRA even though through the different pathways, suggesting multiple targets for the treatment of asthma.

In conclusion, our data demonstrated the effect of GBFXD on airway remodeling by regulating the ECM proteins in CRA mice. The potential mechanisms may be related to inhibiting RTN4 and subsequently suppressing the activation of the PI3K/AKT signaling pathway to reduce the deposition of ECM related molecules. This study provides a new insight into the mechanism of GBFXD on improving airway remodeling for the treatment of CRA.

## Data Availability

The mass spectrometry proteomics data have been deposited to the ProteomeX change Consortium via the PRIDE partner repository with the dataset identifier PXD020711.
