# Supplementary figures and images for: iTRAQ-Based Proteomics Reveals Gu-Ben-Fang-Xiao Decoction Alleviates Airway Remodeling via Reducing Extracellular Matrix Deposition in a Murine Model of Chronic Remission Asthma
Source: Front Pharmacol. 2021 Jun 14;12:588588. doi: 10.3389/fphar.2021.588588 (PMC8237094; doi:10.3389/fphar.2021.588588)

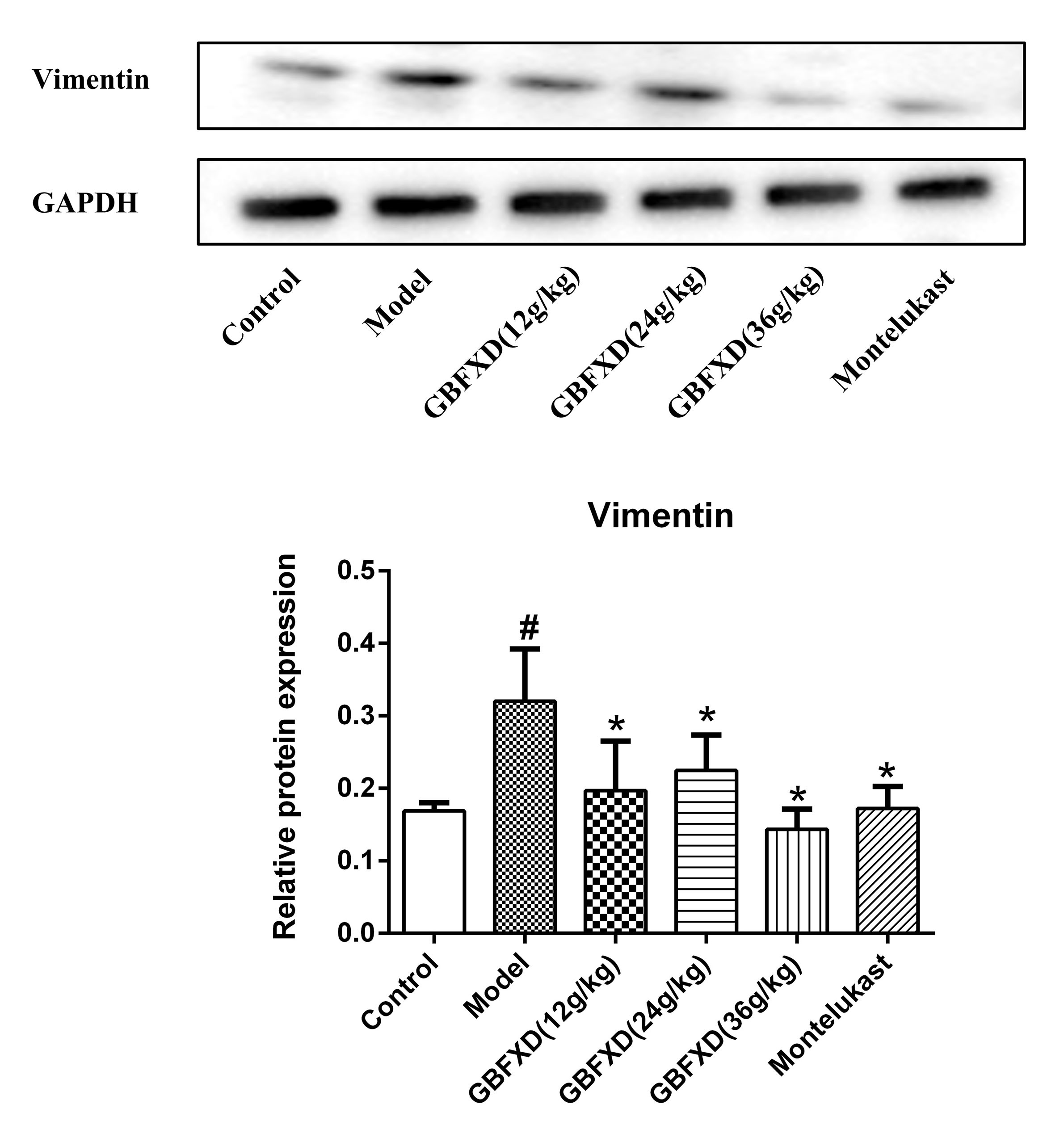

Supplement: Supplementary file 1 [file image1.jpeg]
